# The role of mitochondrial proteases in inflammation and immunity

**DOI:** 10.3389/fimmu.2026.1761658

**Published:** 2026-06-23

**Authors:** Anna Rebeka Oliveira Ferreira, Emily A. Day

**Affiliations:** Department of Physiology and Pharmacology, Schulich School of Medicine and Dentistry, The University of Western Ontario, London, ON, Canada

**Keywords:** cGAS-STING, immune cells, inflammatory disease, innate immunity, macrophages, MAVS, mitochondrial dysfunction, mtDNA

## Abstract

The global rise in chronic inflammatory and autoimmune disorders has intensified research to understand cellular stress response pathways that drive immune dysregulation. Mitochondria have emerged not only as central hubs of cellular metabolism but also as active modulators of immunity and inflammation. Mitochondrial proteases are essential regulators of mitochondrial protein quality control, dynamics, and stress responses. By selectively degrading misfolded or damaged proteins, they maintain mitochondrial function and bioenergetic capacity. Beyond housekeeping roles, mitochondrial proteases also influence immune signaling by modulating mitochondrial stress pathways, reactive oxygen species production, and the release of mitochondrial-derived danger signals. Dysregulation of these proteases has been linked to chronic inflammation and contributes to the pathogenesis of inflammatory diseases. This review summarizes current knowledge on the role of mitochondrial proteases CLPXP, LONP1, i-AAA, m-AAA, as well as processing peptidase OMA1, in immune cells and inflammatory pathologies. We explore the molecular mechanisms by which these mitochondrial proteases regulate immune signaling, integrating the results from immune cells as well as other non-immune cell types, including those involved in cancer, neurodegeneration, renal injury, and other inflammatory pathologies. We explore mitochondrial proteases function as context-dependent regulators of immunometabolic signaling, with effects shaped by cell type, metabolic state, and stress conditions. Finally, we discuss emerging small molecules and drugs targeting mitochondrial proteases to highlight their potential therapeutic role in modulating inflammation. By situating mitochondrial proteases at the crossroads of immunometabolism and therapeutic intervention, this review underscores their untapped potential in the development of innovative anti-inflammatory strategies.

## 1 Introduction

Chronic inflammatory and autoimmune diseases represent a growing global health burden (1), yet the molecular mechanisms that initiate and sustain dysregulated immune responses remain incompletely understood. Recent studies have revealed that cellular metabolism and mitochondrial function play a central role in shaping immune cell behavior (2). Mitochondria have long been recognized for their fundamental role in ATP production, often described as the “powerhouse of the cell, ” a metaphor arising from pioneering discoveries of how mitochondria generate energy through oxidative phosphorylation (OxPhos) (3, 4). Beyond energy production, mitochondria are increasingly appreciated as central integrators of metabolic and signaling networks, coordinating the interplay between cellular metabolism and immune responses. By sensing metabolic signals, mitochondria modulate inflammatory pathways that, in turn, influence cellular differentiation, function, and overall tissue homeostasis across diverse cell types and tissues (5).

**Figure 1:**
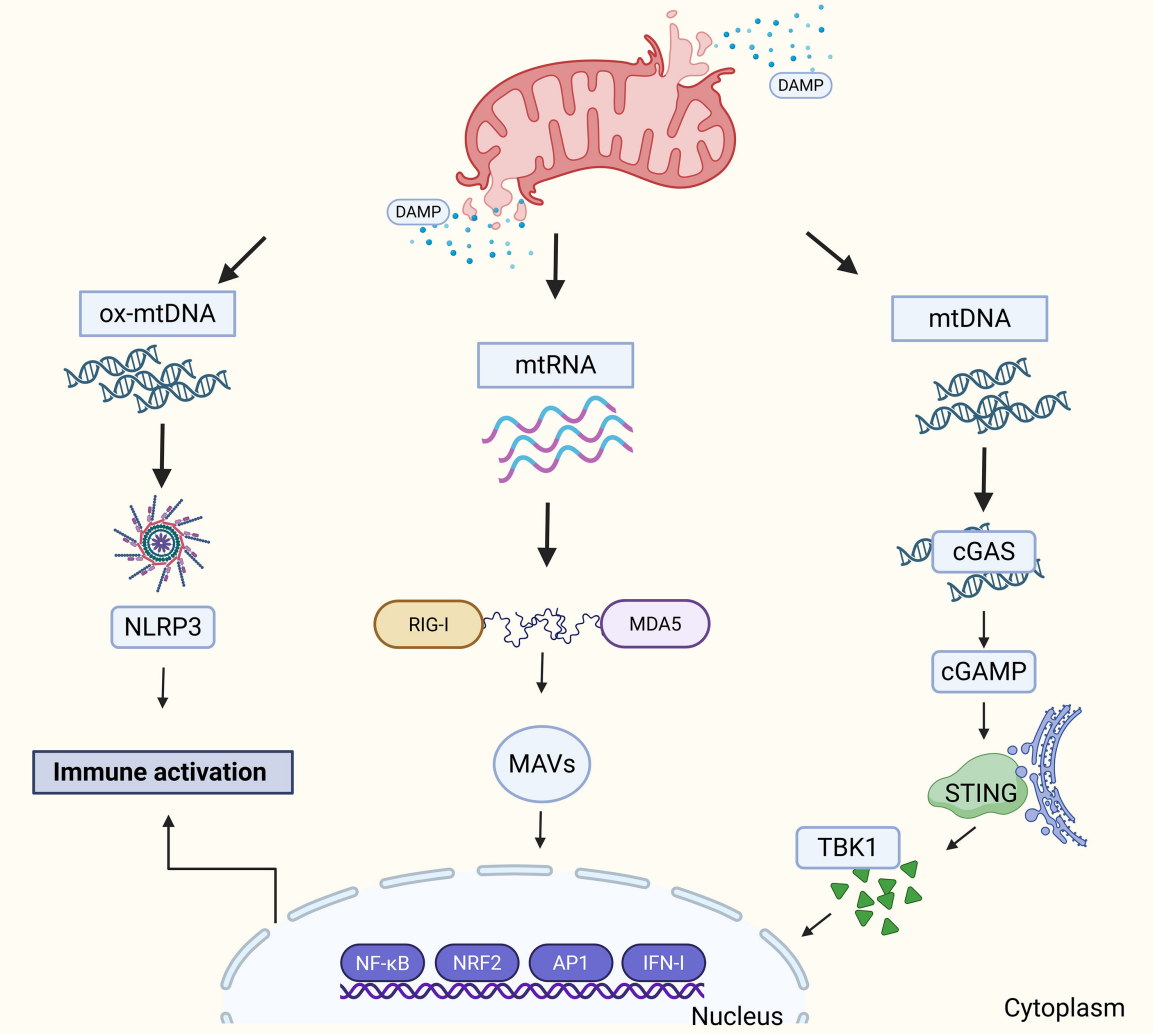
Mitochondrial stress-induced inflammatory signaling. Mitochondrial dysfunction or stress results in the release of DAMPs into the cytosol, including ox-mtDNA, mtRNA, and mtDNA fragments. Ox-mtDNA is sensed in the cytoplasm and initiates assembly of the NLRP3 inflammasome, leading to recruitment of ASC and pro-caspase-1 and the subsequent maturation and secretion of pro-inflammatory cytokines, such as IL-1β and IL-18, driving innate immune activation. mtRNA is sensed by the cytosolic RNA sensors RIG-I and MDA5, which signal through the mitochondrial adaptor MAVS, promoting activation of downstream kinases and transcription factors, including NF-κB and AP-1, to induce pro-inflammatory pathways. In addition, cytosolic mtDNA binds to cGAS, leading to the synthesis of cGAMP, which activates STING on the ER and Golgi membranes. Activated STING recruits and stimulates TBK1, which phosphorylates transcription factors such as IRF3, driving their translocation into the nucleus and the expression of IFN-I. Created with BioRender.com.

Mitochondrial physiology encompasses processes from oxidative phosphorylation to the generation of mitochondrial nucleic acids, both of which directly impact innate immunity (2). It has also been proposed that, due to their prokaryotic ancestry, mitochondria harbour molecular patterns, such as nucleic acids, lipids, and proteins that, when released into the cytosol, can trigger inflammatory responses (6). They retain genomic, structural, and biochemical features reminiscent of their bacterial ancestors, including mitochondrial DNA (mtDNA), double-stranded RNA (mtRNA), and lipids like cardiolipin, which when exposed during organelle damage, or cellular stress can activate innate immune receptors (7). Cytosolic sensors such as cGAS-STING, RIG-I/MAVS, and inflammasomes recognize these Danger-associated molecular patterns (DAMPS) (**Figure 1**), which results in inflammatory signaling cascades that lead to the production of type I interferons (IFN-1), other pro-inflammatory cytokines, and the activation of downstream immune responses (8). Mitochondrial-derived signaling thus constitutes a critical link between cellular stress and innate immune activation.

In addition to their prokaryotic origins triggering immune responses, mitochondria undergo extensive metabolic remodeling upon immune cell activation (9–11). In many innate and adaptive immune cells, inflammatory stimulation leads to rewiring of the tricarboxylic acid (TCA) cycle and altered flux through key metabolic pathways (12, 13). While some metabolites, such as itaconate (14), are produced predominantly by myeloid cells, particularly macrophages, other metabolites, including succinate (15, 16) and fumarate (17), accumulate more broadly during immune activation, as cells shift toward glycolysis and TCA cycle activity becomes disrupted (18, 19). These metabolites can also influence mitochondrial respiration, membrane potential, and the activity of electron transport chain complexes, thus affecting mitochondrial function (20). Succinate accumulates as a result of reduced succinate dehydrogenase activity (SDH, complex II of the electron transport chain) (19) or through alternative pathways, such as glutamine-dependent anaplerosis or the GABA shunt, which provide additional substrate (16, 19). Fumarate can also accumulate through impaired SDH activity (21) via the purine nucleotide and malate/aspartate cycles (18), or reductions in fumarate hydratase abundance, and increases in the aspartate-argininosuccinate (AAS) shunt (17). In the inflammatory context, elevated succinate levels can enhance electron input into the electron transport chain, which, together with a high mitochondrial membrane potential, promotes reverse electron transport at complex I and drives the production of mitochondrial reactive oxygen species (17, 22). In addition to this, during the immune activation, cells shift toward glycolytic ATP production, which limits the use of oxidative phosphorylation and increases the mitochondrial membrane potential (15).

During inflammation, the mitochondrial network also undergoes morphological remodeling, due to increased mitochondrial fission and decreased fusion (23). Excessive fission promotes mitochondrial fragmentation and the release of ROS and DAMPs, amplifying inflammatory signaling; also, impaired fusion compromises bioenergetics and stress adaptation, further shaping the inflammatory phenotype of immune cells (24). Indeed, in models where only mitochondrial morphology is altered (i.e. blocking fission or fusion), these changes are sufficient to drive inflammatory cytokine production in the absence of classical inflammatory signals (25) and can augment cytokine production in response to inflammatory challenges (26). Altered mitochondrial activity during immune activation is also accompanied by changes in mitochondrial-derived ROS (27). These are critical signaling intermediates that modulate cellular responses through effects on transcriptional regulation and cytokine production (28), antimicrobial defense programs, and resolution pathways (29, 30). Under stress conditions, such as inflammation or cellular injury, ROS levels increase and function as secondary messengers that initiate several signaling cascades. Through the oxidation of key signaling molecules, ROS can activate transcription factors, including NF-κB, AP-1, and NRF2, which in turn modulate the expression of genes involved in inflammation, immune responses, cell survival, and oxidative stress adaptation (31–33). ROS further promote the activation of cytokine gene transcription, driving the release of pro-inflammatory cytokines like TNF-α, IL-1β, and IL-6, which play central roles in immune cell recruitment and the amplification of inflammatory pathways (34, 35). While changes in mitochondrial oxidative phosphorylation, ROS production and TCA cycle metabolism have been extensively studied in the context of immune activation and inflammatory responses, the complete scope of mitochondrial signaling networks is still being elucidated.

Mitochondrial proteases are specialized enzymes located within different mitochondrial compartments that maintain protein quality control by degrading misfolded, damaged, or unassembled proteins, thereby preserving mitochondrial function and cellular homeostasis. By sensing and mitigating mitochondrial stress, these enzymes maintain the balance between quality-control processes and the controlled release of signaling molecules that communicate organellar status to the cell (36). In this review, we discuss current knowledge on ATP-dependent mitochondrial proteases, including CLPXP, LONP1, m-AAA, iAAA, as well as processing peptidase OMA1 and their contributions to immune regulation and inflammatory disease. We discuss how mitochondrial proteases actively shape inflammatory responses in immune cells and other cell types across several disease models. We further highlight the therapeutic potential and challenges of targeting these proteases as a novel class of anti-inflammatory interventions (**Table 1**). Overall, this review demonstrates mitochondrial proteostasis as a central regulator of immune homeostasis and a potential target in chronic inflammatory disease.

**Table 1:** Small molecule modulators of mitochondrial proteases and their mitochondrial and inflammatory effects.

Target	Compound	Cell types/mouse model	Key results	Caveat/concerns
LONP1	Bortezomib (37)	THP-1 + oxLDL	↑ Mitochondrial membrane potential	Also targets the 26S proteasome Risk of peripheral neuropathy and renal toxicity (38, 39)
		THP-1 foamy macrophages	↑ Mean fluorescence intensity of GPX4 and mtTFA in BODIPY^+^ CD68^+^ cells	
		ApoE^-^/^-^ mice + HFD + Iron	↓ Aortic plaque area and necrotic core area; ↑ MiniFCT: no significant change in lipid profile, plaque lipid content, or collagen in the aortic sinus	
	CDDO (11)	BMDM + palmitate	↑ p62 and Parkin expression ↓ IL-1β levels ↓ IL-1β and Caspase-1 expression	Also targets: Keap1 (40, 41), IKK-β (42), JAK1, and STAT3 (43) Risk of cardiovascular events (44)
	WSTLZTD (45)	Senescent BMDMs/Raw 264.7 + H_2_O_2_	↓ P16, P21 and F4/80 expression ↓ β-Gal activity ↓ cGAS and STING expression ↑ LONP1 expression	Herbal mixture; target assignment to LONP1 remains provisional Caveat Limited clinical studies in humans; Genetic validation of its targets is still required
		Senile osteoporosis mouse model (SOP)	↓ IL1β and IL6 levels	
	LONP1-IN2 (45)	BMSCs treated with conditioned medium from RAW264.7	↑ cGAS and STING expression	Limited studies using this compound, can also target the 20S proteasome at high concentrations (IC_50_ >10µM) (46)
OMA1	BTM-3566 (47, 48)	Various Cancer Cell Lines	↑ DELE1 cleavage ↑ Apoptosis (BAX-dependent, caspase-3/7) ↑ ATF4 expression/ISR activation	Limited evidence in non-malignant cells
OPA1	MYLS22 (49)	BV2 Cells and HIBD model	↓ OPA1 expression ↓ Mitochondrial membrane potential ↑ Microglial activation ↑ NLRP3, ASC, Caspase-1, GSDMD, IL-1β, IL-18 expression in microglia ↓ Neuronal survival/integrity	Broad Mitochondrial cristae disruption (50)
OPA1	Empagliflozin (51)	BV2 microglial cell line	↓ OPA1 cleavage, mitochondrial fusion and neutralizing mtROS production Genetic ablation of OPA1 abolished the anti-inflammatory effects of empagliflozin in this model	Primary target SGLT2 Empagliflozin does not directly target OPA1, but its downstream consequences are mediated via OPA1 activity in this model
YME1L	NSC319726 (52)	Pharmacogenetic database	Promotes expression of YME1L1	Expression data only, no functional data
	SB216763 (52)	Pharmacogenetic database	Suppresses YME1L1 expression	Expression data only, no functional data
ClpP	NCA029 (53)	CD4^+^ T cells + DSS	↑ ClpP expression ↓ OXPHOS, ↓ OCR mitochondrial respiration ↑ Glycolytic enzymes expression (HK-2, LDHA)	Highly selective and potent, but a narrow therapeutic window due to cytotoxic effects. Downstream effects of ClpP activation are wide-ranging and cell-type and dose-dependent
		CD4^+^ T cells	↓ STAT3 phosphorylation and mRNA ↓ IL-17 and IL-21 levels in Th17 cells ↑ TGFβ and IL-10 levels in Treg cells	
		Colitis mouse models	↓ TNF-α, IL-6, and IL-1β levels ↓ Inflammatory cells and collagen fibers in the colon tissue ↓ CD86 and ↑CD163 ↑Treg cells and ↓Th17 cells	
	A2-32-01 (54)	MM cells	↓ MM cell and tumor growth ↑ ClpP substrates (OAT) ↓ Polyamine biosynthesis ↑ IFN-l response	Limited *in vivo* data; Off-target effects remain unclear
	7b (55)	MM cells	↑ Mitochondrial matrix degradation ↑ TUNEL-positive cells and Caspase-3 expression ↑ ROS levels ↓ ATP production ↑ PINK1 and Parkin expression	Short half-life in culture (rapid degradation), limited evidence in non-malignant cells
	ZG111 (56)	PDAC cells PDAC xenografts and PDX mice	↓ OXPHOS complexes, cristae structural collapse, and impaired mitochondrial function ↑ Mitochondrial ROS ↓ ATP production ↑ p-JNK, p-c-Jun, ATF3, CHOP expression	Induces broad mitochondrial stress
	A54556A (57)	High-fat diet induced NASH in mice	↓ ROS levels, ↑ OXPHOS function ↓ Hepatic steatosis and fibrosis ↓ TNF-α, IL-6, IL-1β and F4/80 mRNA expression ↓ P-JNK and P-p65 activation	Induces ClpX-independent proteolysis, limited target validation
	ONC201 (42, 58)	Human Triple Negative Breast Cancer (TNBC) cell lines SUM159	↑ ATF4 and CHOP expression	Also targets dopamine receptors (DRD2 and DRD3) (59, 60)
		Human Medulloblastoma cells	↑ Apoptosis and mitochondrial dysfunction ↑ ATF4 expression	
	ONC206 (42)	Human Medulloblastoma cells	↑ ↑ Apoptosis and mitochondrial dysfunction ↑ ATF4 expression ↓TFAM expression Prolongs survival and delays tumor growth in medulloblastoma PDX mouse models	High potency resulting in degradation of non-native substrates. High cytotoxicity (61)

Table legend: Activators are in green and inhibitors in orange.

### 1.1 Mitochondrial protein turnover and canonical roles of mitochondrial proteases

The majority of mitochondrial proteins are encoded by nuclear DNA, and mitochondrial protein biogenesis requires the synthesis of precursor proteins in the cytosol, followed by their import into the organelle (62). Most precursor proteins possess an N-terminal presequence that is cleaved upon import to obtain functional mitochondrial proteins (63, 64). Processing by the mitochondrial processing protease (MPP), which removes the presequence, is often followed by a second cleavage event by the mitochondrial intermediate peptidase (MIP) (64, 65). Peptides accumulating in mitochondria are not only derived from presequence cleavage but also from the degradation of nonfunctional, or aged, mitochondrial proteins. Additional mitochondrial proteases coordinate protein turnover via their proteolytic activity. Once considered nonspecific degraders of damaged proteins, it is now recognized that mitochondrial proteases also regulate mitochondrial function through targeted degradation of specific substrates, regulating mitochondrial structure and metabolism and through chaperone and protein binding activities (66). Through these processes, mitochondrial proteases regulate signaling as well as the homeostasis of mitochondria, thereby limiting the release of mitochondrial DAMPS such as mtDNA (67), mtRNA, cardiolipin (68), as well as excess ion/metabolite release such as calcium (69) or ATP (70). In this context, the mitochondrial protein processing regulates organelle function and also modulates innate immune signaling, highlighting mitochondria as key regulators of sterile inflammatory responses. This connects mitochondrial protein processing directly to sterile inflammation, defined as activation of the innate immune system in the absence of pathogens in response to tissue damage, ischemia, chemical injury, or other danger signals (71).

**Figure 2:**
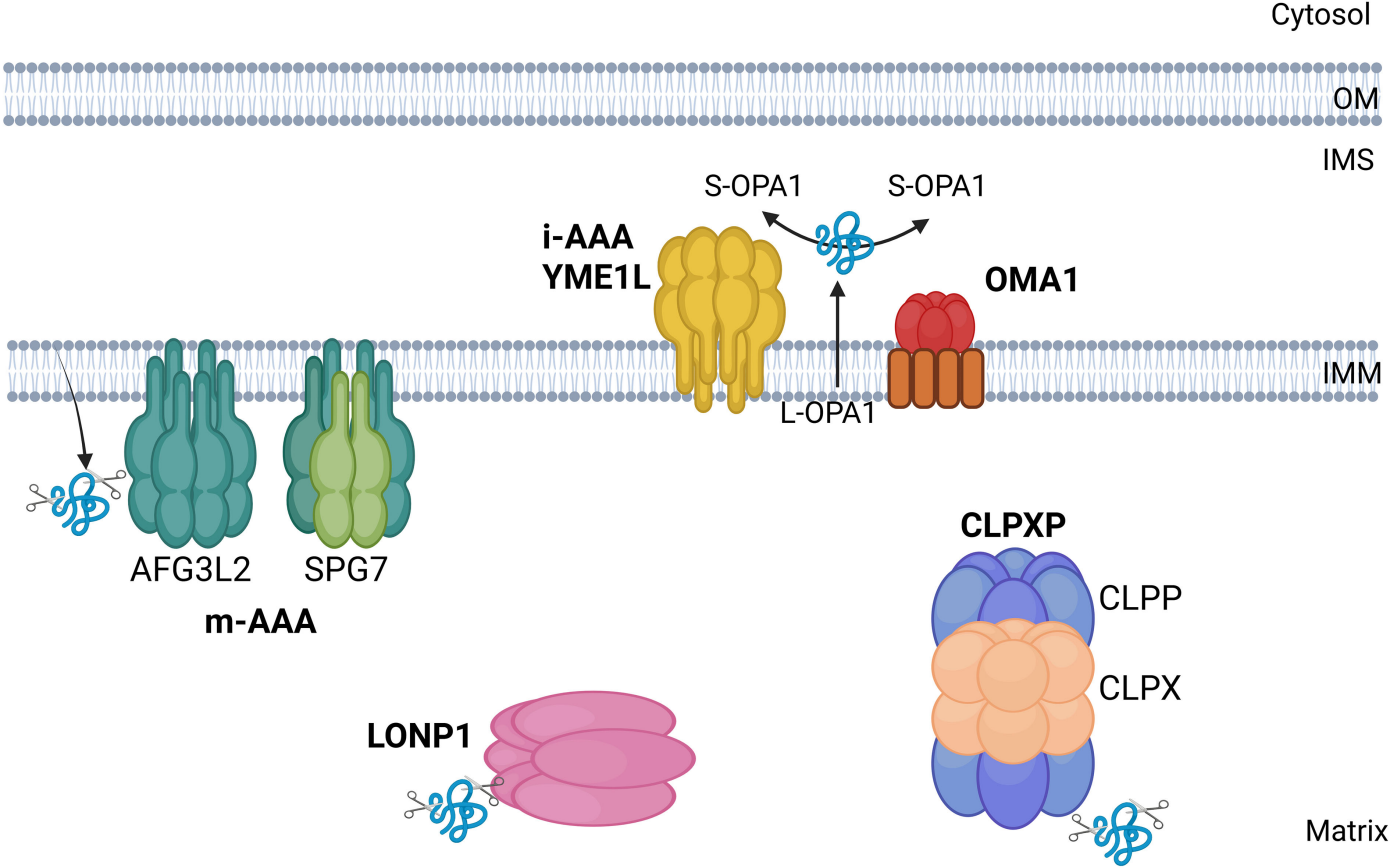
Mitochondrial proteases as regulators of mitochondrial function. Mitochondrial matrix and inner membrane (IM) proteases maintain protein quality control. In the matrix, LONP1 degrades misfolded or damaged proteins, while the CLPXP complex, composed of the proteolytic subunit ClpP and the ATPase/chaperone CLPX, mediates degradation of matrix proteins and substrate recognition. Within the IM, the m-AAA proteases have their catalytic sites facing the matrix. They assemble as either homo-oligomers of AFG3L2 or hetero-oligomers of AFG3L2 and SPG7, and are responsible for degrading damaged IM proteins and processing regulatory proteins to maintain mitochondrial function and integrity. The i-AAA protease, composed of six subunits of YME1L with catalytic sites facing the intermembrane space (IMS), extracts and degrades IM proteins and processes the long form of OPA1 (L-OPA1) to generate the short form (S-OPA1). OMA1, another IM protease with catalytic activity toward the IMS, also cleaves L-OPA1 under stress conditions, contributing to mitochondrial fusion regulation and cristae remodeling. Created with BioRender.com.

In the mitochondria, protein homeostasis is regulated by mitochondrial proteases, including four core ATP-dependent proteases, the m-AAA protease (composed of AFG3L2 and SPG7), the iAAA protease (containing YME1L1), LONP1, and CLPXP (composed of CLPP for proteolysis and CLPX for substrate recognition) (65, 72). The m-AAA protease’s catalytic domain is oriented toward the mitochondrial matrix, whereas the i-AAA protease’s catalytic domain faces the intermembrane space. In contrast, the matrix-resident proteases LONP1 and CLPXP are localized entirely within the mitochondrial matrix (**Figure 2**). Their mechanism of action is evolutionarily conserved and involves an ATP-dependent unfolding of damaged or misfolded proteins by the AAA^+^ domain, followed by transfer into the proteolytic core for degradation (65, 72). Other mitochondrial proteases, including OMA1, PARL, and X-pro aminopeptidase 3 (XNPEP3), are localized to the inner membrane and respond to diverse stimuli, such as loss of mitochondrial membrane potential (73). Notably, both OMA1 and the i-AAA protease regulate OPA1, thereby modulating mitochondrial fission and fusion (73). In this review, we focus primarily on ATP-dependent proteases, but we also discuss OMA1 due to its functional overlap with i-AAA in controlling mitochondrial dynamics.

## 2 CLPXP

The CLPXP complex is localized in the mitochondrial matrix and formed by the ATP-driven unfoldase ClpX and the proteolytic core ClpP. ClpP is a serine protease that oligomerizes into heptameric rings, which then stack to form a tetradecameric double-ring structure, containing a proteolytic chamber where substrate degradation occurs (74). Within this chamber, cleavage results in a sequential and processive hydrolysis of protein substrates, ensuring efficient degradation of misfolded or damaged proteins (75). Substrate entry into the ClpP chamber is regulated by the AAA+ unfoldase ClpX, which recognizes specific degradation tags, unfolds the substrate, and translocates it into ClpP for proteolysis. In Escherichia coli ClpXP, degrades proteins bearing a C-terminal degron called the ssrA tag that is added during tmRNA-rescue of stalled ribosomes (76, 77), however, in eukaryotic cells, the tagging and substrate recognition are less well understood, although serine phosphorylation seems to increase targeting to CLpX via binding to the recognition loop (RKL loop) in ClpX (78). Thus, eukaryotic CLPXP is currently thought to largely target damaged, misfolded, or unassembled proteins. Several bona fide substrates of CLPXP have been identified, including HSPA9, Ornithine Aminotransferase and subunits of Complex I and Complex II of the ETC. Thus, CLPXP regulates the turnover of key mitochondrial proteins, thereby maintaining respiratory chain integrity and sustaining oxidative phosphorylation (79). Therefore, CLPXP is essential for the surveillance and maintenance of mitochondrial quality control. As a result, the functional outcomes of CLPXP activity range from pro-inflammatory to anti-inflammatory and are modulated by multiple determinants, including cell lineage, metabolic status, and mitochondrial stress.

ClpP loss triggers innate immune signaling in a cell type–specific manner, with key differences seen between dividing and non-dividing cells. In postmitotic neurons of aged ClpP^−/−^ mouse brains, loss of ClpP results in chronic disruption of the mitochondrial unfolded protein response (mtUPR), leading to accumulation of STAT1 and the co-chaperone DNAJA3 in mitochondrial and nuclear compartments, triggering sterile innate immune activation. In 3-month-old ClpP-null brains, STAT1, DDX58, and interferon-stimulated gene 15 (ISG15) were elevated, indicating that sterile inflammation precedes the neurodegenerative phenotypes (80, 81). In 11–19-month-old brains, these proteins were further increased, correlating with progressive neuroinflammation and neurodegeneration. Notably, other innate immune factors, including IRF3 and IRF7, several NFκB-associated proteins, and TLR9, did not show significant induction, suggesting that ClpP deficiency selectively activates specific inflammatory pathways rather than inducing a global immune response. This targeted, chronic, sterile inflammatory state may contribute to the late-onset neurodegeneration observed in ClpP silencing (80).

In contrast, in models that are used to study mitochondrial encephalopathies and neurodegeneration, such as DARS2-deficient mice, loss of ClpP appears to mitigate inflammation, preserving mitochondrial integrity and reducing neurodegeneration. Compared to DARS2 knockout mice with intact ClpP, which show extensive microglial activation, an increase in IBA-1 levels, and reactive microglial morphology, DARS2-KO mice with ClpP deletion show fewer activated microglia, lower IBA-1 levels, and preservation of microglial morphology, correlating with delayed neuronal degeneration. Mechanistically, ClpP loss stabilized the Complex I N-module, preserved mitochondrial morphology, and maintained OXPHOS function in neurons, reducing apoptosis and preventing the release of pro-inflammatory danger signals (82, 83). As a result, microglial recruitment and activation were suppressed. These results suggest that inhibiting ClpP may protect against neurodegeneration, which may be associated with a decrease in inflammation in mitochondrial encephalopathies (83). Together, these studies suggest that the functional impact of ClpP is context dependent, shaped by metabolic state, stress conditions, and mitochondrial health; its loss is detrimental under normal mitochondrial conditions, yet protective in the setting of mitochondrial disease.

In the context of inflammatory diseases, ClpP expression is elevated in colonic biopsies in both active and inactive stages of ulcerative colitis and Crohn’s disease compared to healthy controls. ClpP expression has also been correlated with disease activity indices and therapeutic responses to infliximab, suggesting a role in modulating remission outcomes. Single-cell transcriptomic profiling further identified that ClpP upregulation occurs in activated CD4^+^ T cells, inflammatory monocytes, macrophages, regulatory T (Treg) cells, and follicular B cells, implicating it in the regulation of mucosal immune responses during intestinal inflammation (53). ClpP activation by NCA029 alleviated IBD severity, reducing weight loss, lessening colon shortening, lowering disease activity index(DAI) scores, and improving survival, effects that were dependent on CD4^+^ T cells. Proteomic analyses revealed that NCA029 downregulates electron transport chain (ETC) subunits, inhibits OXPHOS, and limits ATP production, reprogramming CD4^+^ T cell metabolism, and therefore limits the proliferation of pro-inflammatory Th17 cells while supporting the expansion and differentiation of Treg cells (53). *In vivo*, NCA029 demonstrated robust efficacy in both DSS-induced acute colitis and IL-10 knockout chronic colitis mouse models. In DSS-induced acute colitis, NCA029 dose-dependently reduced serum pro-inflammatory cytokines, including TNF-α, IL-6, and IL-1β, and mitigated mucosal infiltration of inflammatory cells and collagen deposition. Treatment shifted macrophage polarization from pro-inflammatory M1 (CD86^+^) to anti-inflammatory M2 (CD163^+^), decreased neutrophils and IgG^+^ plasma cells, and promoted recovery of tight junction proteins ZO-1, claudin-1, and occludin, thereby enhancing intestinal barrier function, restoring epithelial proliferation, redistributing CD263, and reducing apoptosis in colonic tissue (53). Similar protective effects were observed in IL-10 knockout mice, where NCA029 increased colon length, alleviated weight loss, reduced inflammatory factor release, and upregulated tight junction proteins. CD4^+^ T cell depletion and reconstitution experiments confirmed that NCA029’s effects depend on ClpP, as ClpP-deficient CD4^+^ T cells were resistant to NCA029-mediated attenuation of disease progression. These results demonstrate that NCA029 mitigates IBD by modulating both innate and adaptive immunity, reducing inflammation, and restoring intestinal homeostasis through cellular and metabolic regulation (53).

Consistent with activation of ClpP being anti-inflammatory, ClpP deficiency has been shown to disrupt mitochondrial DNA integrity and lead to cytosolic mtDNA release (84) and activation of the cGAS–STING–IFN-I pathway, which establishes a potent antiviral state (85). ClpP-KO mouse embryonic fibroblasts (MEFs) have an elevated baseline expression of ISGs, enhanced inducible antiviral responses, and strong resistance to both RNA (VSV) and DNA (HSV-1) viruses compared to wild-type cells. Double Knockout (DKO) of ClpP and STING confirm STING-dependence, as effects were abolished in the DKOs. Mechanistically, ClpP-KO cells show mtDNA nucleoid enlargement and aggregation, which can be reversed by inhibiting mtDNA replication (using small molecule ddC or *Polg2* knockdown), reducing ISG expression. Blocking mtDNA release with VBIT-4 similarly lowers baseline ISG levels, indicating that cytosolic mtDNA is the key trigger. However, it remains unclear whether ClpP directly controls mtDNA containment or whether cytosolic mtDNA release occurs indirectly, as a secondary consequence of nucleotide imbalance. This phenomenon is conserved in human fibroblasts, where ClpP reduction increases antiviral ISGs and STAT proteins (IFIT2, IFITM1, RIG-I, STAT1, STAT2). Modulation of mitochondrial chaperones (CLPX), fission/fusion proteins (Mfn1, Drp1), or mitochondrial translation (chloramphenicol) does not affect ISG induction, highlighting the central role of ClpP in regulating mtDNA-driven innate antiviral defenses. This suggests that targeting ClpP or its downstream pathway could be a strategy for antiviral or immunotherapeutic interventions (86).

Similarly, ClpP knockdown in multiple myeloma (MM) cells triggers a robust IFN-I response driven by mitochondrial dysfunction and cytosolic DNA sensing. RNA-seq revealed strong upregulation of IFN-α/IFN-γ–related pathways and ISGs, which was confirmed by increased TBK1 phosphorylation, elevated ISG expression, and higher IFN-β production in both inducible knockdown models and upon pharmacologic ClpP inhibition (A2-32-01). Studies also demonstrate that human MM cells have an increase in ClpP expression (54, 55). ClpP knockdown impaired cell growth, induced apoptosis, disrupted mitochondrial structure and OXPHOS, reduced ATP production, increased ROS, and sensitized cells to standard therapies (54, 55). Activation of ClpP with the ClpP agonist 7b induces extensive degradation of respiratory chain proteins, triggers PINK1/Parkin-dependent mitophagy, and drives apoptosis in MM *in vitro* and *in vivo (*55). In contrast, the pharmacologic inhibition of ClpP using A2-32–01 shows accumulation of ClpP substrates and reduces MM cell growth both in cultured cell lines, in primary CD138^+^ patient samples and in murine *in vivo* models (54). Notably, these seemingly opposing effects of ClpP activation and inhibition within the same disease model remain unclear but likely reflect the need for tightly balanced protease activity, where both excessive and insufficient ClpP function disrupt mitochondrial proteostasis and impair cellular survival. Future studies are needed to determine whether differences in experimental context, substrate specificity, or the mitochondrial stress levels account for the apparent contradiction.

Beyond its cell-intrinsic role, ClpP silencing in MM cells also stimulates a potent antitumor immune response *in vivo*. The enhanced IFN-I response enhances the immunogenicity of MM cells, increasing surface HLA and CD38 expression (87) and producing cytokines that activate dendritic cells (DCs), boosting their ability to prime T cells. In the immunocompetent KaLwRij mouse model, intravenous injection of ClpP-silenced 5TGM1 cells significantly prolonged animal survival and reduced bone marrow (BM) infiltration compared with controls, coinciding with sustained IFN-I pathway activation. This immune-mediated tumor containment was associated with increased IFN-γ, CD4^+^ and CD8^+^ T cells, higher levels of central and effector memory CD4^+^ T-cell subsets, and reduced proportions of exhausted CD4^+^ T cells (54). In contrast, the ClpP knockdown did not impair 5TGM1 proliferation *in vitro* or delay tumor formation in immunodeficient NSG mice. These results indicate that ClpP inhibition promotes effective antimyeloma immunity by reshaping the BM immune microenvironment toward a more activated and less immunosuppressive state, suggesting that targeting ClpP could complement immunotherapies and represents a promising strategy for metabolic reprogramming in MM treatment (54).

In pancreatic ductal adenocarcinoma (PDAC) cells, ZG111, a small-molecule ClpP activator, enhances ClpP proteolytic activity, induces degradation of mitochondrial respiratory chain complexes, triggers mitochondrial stress, and activates the JNK/c-Jun pathway and ER stress response, suppressing tumor cell proliferation (56). In addition, the activation of mitochondrial stress and JNK/c-Jun signaling plays a critical role in immune cell function, including T cell activation, proliferation, and cytokine production, as well as NK cell cytotoxicity and macrophage polarization (88). In contrast, in hepatocytes under lipotoxic stress, ClpP downregulation drives mitochondrial dysfunction, increases ROS, and activates stress/inflammatory pathways, including P-JNK, P-NF-κB p65, cGAS-STING signaling, and pro-inflammatory cytokine expression, which contributes to hepatic inflammation and fibrosis in NASH models. Pharmacologic or genetic restoration of ClpP (via A54556A compound or AAV-ClpP) suppresses these inflammatory signals, reduces cytokine production, improves mitochondrial function, and protects against NASH progression (57). Overall, these findings suggest that the functional impact of ClpP is context-dependent; its activation is detrimental in rapidly dividing cancer cells but protective in lipotoxic stress.

Several other small molecules have been developed to modulate the mitochondrial protease ClpP, highlighting its potential as a therapeutic target in cancer and other diseases. ONC201, ONC206, and ONC212 are imipridone derivatives that activate ClpP. These compounds impair mitochondrial function, decrease respiratory chain proteins, and trigger the mtUPR and apoptosis in cancer cells (89), particularly in multiple myeloma (90), lymphocytic leukemia (91), glioblastoma (92, 93), and hepatocellular carcinoma (40). ONC201 was recently approved for patients with recurrent H3 K27 M-mutant diffuse glioma, while a Phase 3 confirmatory trial (ACTION - NCT05580562) is ongoing in newly diagnosed disease (41, 94). ONC206 is currently in Phase I clinical trials for pediatric patients with primary brain tumors (42). ClpP-activating compounds such as ClpP1071, ClpP2068, and 7K also explore this mechanism, demonstrating selective cytotoxicity in tumor cells with high ClpP expression in hematologic cancers (43, 95, 96). These results indicate that pharmacologic hyperactivation of ClpP induces tumor cell apoptosis, engages resistance pathways, and represents a novel strategy to target mitochondrial proteostasis across multiple cancers. Given ClpP’s emerging roles in immune and inflammatory signaling, these agents or next-generation derivatives may also be evaluated at lower, non-cytotoxic doses to modulate mitochondrial stress pathways in inflammatory diseases. Such repurposing could reveal new therapeutic windows distinct from their antitumor activity.

## 3 LONP1

LONP1 is an ATP-dependent protease localized primarily in the mitochondrial matrix of eukaryotic cells, although recent studies have also detected its presence in the nucleus, cytosol, and endoplasmic reticulum (97, 98). It is synthesized in the cytosol as a precursor protein containing an N-terminal mitochondrial targeting sequence (MTS), which is cleaved upon import into mitochondria to produce the mature form (99, 100). LONP1 has three distinct domain structures: an N-terminal domain (NTD) for substrate recognition, a central AAA+ ATPase domain responsible for ATP binding, unfolding the substrate protein, and a C-terminal protease domain containing a conserved serine–lysine dyad that forms the catalytic site. The canonical roles in proteostasis of LONP1 are supported by its structural organization, which enables coupling of ATP hydrolysis with proteolytic activity, ensuring efficient degradation of misfolded or damaged mitochondrial proteins (98). LONP1 also functions as a molecular chaperone and DNA-binding protein, contributing to the maintenance of mitochondrial transcription and the regulation of key metabolic enzymes (101, 102). While a specific consensus sequence recognized by LONP1 has not been described, it preferentially binds proteins with exposed hydrophobic regions, a hallmark of misfolding or damage. Structural studies further indicate that the NTD plays a critical role in substrate recognition and is a key determinant of LONP1 activation (103). Previous studies have shown several LONP1 substrates, including aconitase (ACO) (104), uracil-DNA glycosylase isoform 1 (105), cystathionine β-synthase (106), mitochondrial calpain 10 (107), DELE1 (108), TFAM (67) and PINK1 (109, 110).

Through its protease activity, LONP1 plays a central role in the integrated stress response (ISR), both by modulating the DELE1–eIF2α–ATF4 signaling axis and being positively regulated by this pathway. Under physiological conditions, DELE1 is degraded by LONP1 after mitochondrial import (108). When this process is disrupted, DELE1 accumulates at the mitochondrial surface, resulting in the activation of heme-regulated inhibitor (HRI), a kinase which activates eIF2α. In parallel, endoplasmic reticulum stress can activate PERK, another eIF2α kinase, which phosphorylates eIF2α and results in the upregulation of LONP1 (109). In macrophages exposed to saturated fatty acids, PERK is activated, resulting in eIF2α phosphorylation and the upregulation of LONP1. In this context, PINK1 was reduced, and therefore Parkin-mediated mitophagy was suppressed, resulting in increased mtROS and inflammasome activation. While PINK1 has been shown to be a substrate of LONP1 (110), it is unclear if the effects of LONP1 in these macrophages are via direct degradation of PINK1 or due to changes in overall mitochondrial health. Pharmacological interruption of the ISR, using the PERK inhibitor GSK2606414 or ISRIB (which acts downstream of eIF2α phosphorylation), reduced LONP1 induction and restored PINK1/Parkin-mediated mitophagy, and suppressed mtROS and inflammasome activation (11). Genetic silencing of LONP1 or its pharmacological inhibition (CDDO) stabilized PINK1, promoted Parkin and p62 recruitment to mitochondria, reduced TOM40 expression, and similarly alleviated mtROS production and inflammasome activation. These results indicate that both ISR modulation and direct targeting of LONP1 converge on mitochondrial quality control to mitigate lipid-driven inflammation in atherosclerosis (11). However, it is important to note the specificity limitations of CDDO as a tool for selectively targeting LONP1. In addition to the inhibition of LONP1, CDDO and derivatives have been shown to modify cysteine residues on key inflammatory proteins such as Keap1 (111–113), IKK-β (114), JAK1, and STAT3 (115).

Another key substrate degraded by LONP1 is TFAM, the mitochondrial transcription factor responsible for nucleoid organization, mtDNA packaging, and the initiation of mitochondrial genome replication and transcription (67, 116). In addition to recognizing substrates, LONP1 activity can be modulated by interactions with other proteins, which can inhibit or activate its protease function. Interestingly, LONP1 has been proposed to interact with lactate dehydrogenase B (LDHB) (37) by PLA assays, immunofluorescent colocalization and Co-IP assays. This LONP1:LDHB interaction is suggested to be inactivating of LONP1 based on reduced TFAM levels in LDHB KO’s. This interaction was shown to be important in the context of lipid-laden or atherosclerotic macrophages because overexpression of HMOX1 allows for preferential HMOX1-LDHB binding, releasing LONP1 and promoting TFAM degradation. TFAM degradation by LONP1 was associated with mitochondrial dysfunction, increased susceptibility to ferroptosis and worsening of atherosclerosis, potentially as a downstream consequence of disrupted mitochondrial homeostasis (37). Also, in these models, pharmacological inhibition of LONP1 with bortezomib in ApoE^-^/^-^ mice subjected to a high-fat diet (16 weeks) and iron overload reduced atherosclerotic plaque development (37); however, bortezomib primarily inhibits the proteasome in addition to inhibition of LONP1 and CLPXP (38). Therefore, the specificity of these effects on LONP1 requires further investigation, potentially through the use of specific LONP1 inhibitors that do not target the proteasome or CLPXP.

Similarly, LONP1 has also been implicated in the immunopathogenesis of systemic lupus erythematosus (SLE). In lupus-prone Fcgr2b*^-^/^-^* mice, LONP1 expression is elevated in CD4^+^ T cells, correlating with mitochondrial oxidative stress (10). The increase in LONP1 activity promoted mitochondrial dysfunction and the cytosolic release of mtDNA, which functions as a potent DAMP (116). This aberrant mtDNA release activates the cGAS–STING–TBK1 axis, leading to phosphorylation of TBK1 and induction of IFN-1 signaling (117). Consequently, this signaling cascade amplifies inflammatory cytokine production and drives hyperactivation of CD4^+^ T cells, a hallmark of lupus immunopathology (10). LONP1 knockdown attenuated mtDNA leakage, reduced ROS levels, dampened cGAS–STING activation, and suppressed T-cell activation. How LONP1 regulates mtDNA release, whether it is associated with TFAM degradation, and therefore disrupted nucleoid organization, and mtDNA packaging or as a secondary consequence of general mitochondrial disruption such as cristae collapse, mitophagy defects, ETC injury, or through specific channel opening, such as VDAC, BAX/BAK or MPTP opening, warrants further investigation. However, these results suggest that excessive LONP1 activity can amplify inflammatory signaling in SLE in autoreactive T-cell responses via the cGAS–STING–TBK1 axis (10).

In contrast, emerging evidence from aging-related diseases suggests that LONP1 loss, rather than its elevation, can promote inflammation by impairing mitochondrial quality control. LONP1 expression is reduced in the kidneys of aged humans and mice. Silencing of LONP1 was shown to accelerate mitochondrial dysfunction, indicated by reduced mtDNA, ATP levels, decreased TFAM expression, increased Drp1 expression, disrupted mitochondrial network, mitochondrial swelling, and cristae collapse, indicating impaired mitochondrial integrity and biogenesis rather than direct regulation of mtDNA by LONP1 (118). Overexpression of LONP1 attenuated D-Gal-induced mitochondrial dysfunction, increasing basal respiration, ATP-coupled respiration, and maximal respiration. Overexpression of LONP1 also alleviated renal fibrosis, while silencing of LONP1 was associated with worsened renal fibrosis and an impairment of the kidney’s regeneration capacity (118). Similarly, during pulmonary fibrosis, the loss of LONP1 in alveolar type 2 cells promotes mitochondrial dysfunction, leading to an increase in fibrosis and the activation of aging markers. LONP1 knockdown was also associated with enhanced NFκB signaling (119). In osteoarthritis, LONP1 knockdown in chondrocytes leads to mitochondrial dysfunction, resulting in a decrease in cartilage repair, increased oxidative stress and activation of mitophagy (120). Thus, while elevated LONP1 has been linked to pro-inflammatory activation in some contexts, evidence from aging-related diseases highlights an opposing paradigm in which LONP1 loss exacerbates mitochondrial dysfunction, inflammation, and tissue degeneration.

This dichotomy is further exemplified in senescent macrophages and senile osteoporosis models (SOP), where reduced LONP1 in H_2_O_2_-induced senescent RAW264.7 cells, (a macrophage-like cell line) appears to drive the cGAS–STING activation and the senescence-associated inflammatory phenotype (45). In this context, the Wen-Shen-Tong-Luo-Zhi-Tong Decoction (WSTLZTD), a traditional Chinese herbal formula that has been clinically used in the treatment of senile osteoporosis (SOP) (121, 122), enhanced LONP1 expression and reduced cGAS activation. Molecular docking revealed that 11 active components of WSTLZTD, including Resveratrol, Gastrodin, Icariin, Luteolin, and Quercetin, strongly bind to LONP1, suggesting direct regulation. The WSTLZTD increased LONP1 expression in BMDMs of aged mice. *In vitro*, H_2_O_2_-induced RAW264.7 senescent macrophages treated with WSTLZTD showed suppressed cGAS/STING pathway activation and improved cell viability, indicating alleviation of senescence. Importantly, inhibition of LONP1 with LONP1-IN-2 abolished the anti-senescent and pro-osteogenic effects of WSTLZTD and failed to suppress cGAS/STING pathway activation in BMSCs, which suggests that WSTLZTD exerts its effects through LONP1-mediated attenuation of macrophage senescence and downstream modulation of cGAS–STING signaling (45), although further genetic validation of this is required. While herbal treatments have shown promise in modulating mitochondrial function and inflammation, more studies are needed to fully understand their mechanisms of action. While some bioactive components of WSTLZTD bind to LONP1; the direct interaction and regulatory mechanisms remain unclear. Notably, the use of LONP1-IN2, a compound reported to selectively inhibit LONP1 without significant effects in the 20S proteasome (46) strengthens the interpretation that these effects are LONP1-dependent.

These observations underscore the importance of understanding how pharmacological modulation of LONP1 is achieved, as well as the challenges that may influence its therapeutic translation. The compound CDDO, also known as bardoxolone and its derivatives, CDDO-Me (or bardoxolone methyl), CDDO-anhydride and CDDO-imidazolide, reversibly inhibit LONP1 in a non-competitive mechanism, blocking ATP hydrolysis. These compounds have demonstrated potent anti-cancer and anti-inflammatory activities in preclinical studies (11, 123). Additionally, CDDO and its derivatives were also tested in clinical trials related to type 2 diabetes and chronic kidney disease (CKD); these trials were discontinued due to safety concerns related to cardiovascular adverse events (44). However, in addition to the inhibition of LONP1, CDDO and derivatives have been shown to modify cysteine residues on key inflammatory proteins such as Keap1 (111, 112), IKK-β (114), JAK1, and STAT3 (115). The potential Keap1 interaction is especially compelling in light of NRF2’s central anti-inflammatory role (124). These findings complicate the interpretation of both the anti-inflammatory effects and safety concerns of CDDO and its derivatives, as covalent modification of key inflammatory regulators, rather than direct targeting of LONP1, may underlie their activity. However, LONP1 itself has been shown to directly influence the downstream target of NRF2, HO-1’s abundance through loss- and gain-of-function studies, supporting a role for LONP1 in regulating this NRF2-dependent antioxidant and anti-inflammatory pathway (125). Therefore, while CDDO and derivatives activate NRF2 pathways via interaction with KEAP1 independently of LONP1-dependent, the role of LONP1 to regulate NRF2 (or HO-1) through an alternative mechanism(s) warrants further investigation.

84-B10 is a synthetic 3-phenylglutaric acid derivative identified as a potent activator of LONP1, which was discovered in studies exploring its therapeutic potential in CKD. 84-B10 demonstrates a strong binding affinity for LONP1 through molecular docking and protease activity assays, binding to the catalytic domain of LONP1 and enhancing its proteolytic activity, such as the degradation of TFAM (126). Structural analysis demonstrates key interactions between the 84-B10 and LONP1, including hydrogen bonds and a salt bridge (126). Interestingly, the LONP1 activation 84-B10 has been shown to reduce BAX and IL1β levels in aristolochic acid I (AAI) induced nephrotoxicity. 84-B10 treatment preserved mitochondrial ultrastructure, restored mitochondrial respiration and reduced mitochondrial ROS generation, and these effects were attenuated when LONP1 was partially knocked down (127). Additionally, 84-B10 has been shown to alleviate cisplatin-induced acute kidney injury by limiting mitochondrial damage and oxidative stress in response to cisplatin (128). These data suggest activation of LONP1 may also be anti-inflammatory in the context of nephrotoxicity. Overall, the dual capacity of LONP1 to either amplify or restrain inflammation, depending on cell type, stressor, and disease context, highlights its emerging role as a mitochondrial rheostat in innate and adaptive immunity. Defining the conditions under which LONP1 inhibition or activation is beneficial will be crucial for safely leveraging this pathway in inflammatory, autoimmune, and age-associated diseases.

## 4 m-AAA protease

AFG3L2 is a core subunit of the mitochondrial m-AAA protease, is localized to the inner mitochondrial membrane and serves as a pivotal regulator of proteostasis in this compartment. In human mitochondria, the m-AAA protease assembles either as homohexameric complexes with AFG3L2 or as heterohexameric complexes containing AFG3L2 with its homolog paraplegin (SPG7) (**Figure 2**) (129). The AAA domains exhibit chaperone-like functions that enable selective recognition of misfolded, solvent-exposed regions within membrane proteins (130). For the processing of membrane-associated substrates, initially, the N- and C-termini of the AFG3L2 engage the substrate in the membrane interface. Following initial interaction at the protease surface, substrates are subsequently pulled from the membrane and translocated into the proteolytic chamber for degradation (131, 132). Studies demonstrate that some of the substrates can include MrpL32 (133), cytochrome *c* peroxidase (Ccp1) (134), and COXII (135). Further, AFG3L2-containing proteolytic assemblies also regulate several essential mitochondrial processes, including the biogenesis and quality control of mitochondrial ribosomes (136), and contribute to mitochondrial calcium homeostasis by controlling the stability and assembly of inner membrane ion-transport machinery (132).

AFG3L2 plays a critical role in maintaining OPA1 stability and therefore regulating mitochondrial fusion (137). Under physiological conditions, AFG3L2 limits OMA1 activity by degrading it after transient stress activation, preserving a balanced ratio of long (L-OPA1) and short (S-OPA1) OPA1 isoforms (138). When the m-AAA function is lost or impaired, resulting in the predominance of S-OPA1 isoforms, this leads to mitochondrial fragmentation and loss of cristae structure (137). These structural dysfunctions facilitate the release of mtDAMPs, such as mtDNA and cardiolipin (139), which can activate inflammatory signaling in astrocytes, microglia, and peripheral immune cells (140, 141), which will be discussed in more detail below. In Drosophila models of AFG3L2 deficiency, mitochondrial dysfunction is triggered by the accumulation of misfolded mitochondrial proteins, leading to the activation of the mtUPR. This response is characterized by the upregulation of mitochondrial chaperones such as Hsp60 and Hsc70-5, as well as the activation of autophagy and mitophagy pathways, which help alleviate the stress caused by protein aggregation. Additionally, AFG3L2-deficient mitochondria exhibit a widespread reduction or loss of cristae density, resulting in mitochondrial damage. Furthermore, the absence of AFG3L2 leads to an increase in ubiquitinated proteins, a marker of cellular stress, suggesting that the mitochondrial quality control mechanisms are overwhelmed (142, 143).

In the mitochondrial inner membrane, Prohibitin 1 (PHB1) interacts with the m-AAA protease, which forms either AFG3L2 homohexamers or AFG3L2–SPG7 heterohexamers (144). PHB1 tethers AFG3L2 and SPG7 to restrain their interaction. This regulates the mitochondrial permeability transition pore (mPTP), preventing its opening and maintaining normal mitochondrial function, thereby limiting mtDNA release during inflammatory stress (141). PHB1 has been shown to regulate macrophage function by maintaining mitochondrial integrity and regulating cellular metabolism, thereby modulating inflammatory responses through the M2 polarization (145). In PHBI overexpression, macrophages show reduced ROS production, an increase in mitochondrial membrane potential and the expression of SOD and CAT, preserving mitochondrial integrity. These metabolic improvements correlate directly with enhanced M2 polarization, as evidenced by increased Arg1, CD206, IL-10, and TGF-β expression, along with improved phagocytic capacity (145). In PHB1-deficient HeLa or J774A.1 cells exposed to stressors such as H_2_O_2_ or LPS, loss of PHB1 destabilizes inner mitochondrial membrane organization, allowing SPG7 to enhance its binding with AFG3L2 (141). SPG7 has been implicated in regulating mPTP activity through interactions with pore-associated components such as cyclophilin D (CypD) and VDAC (146). Under stress conditions, this enhanced interaction of AFG3L2 and SPG7 in the absence of PHB1 alters the activity of the m-AAA protease complex and drives pathological mPTP opening through a mechanism that does not depend on SPG7 binding to traditional pore-associated components such as VDAC or cyclophilin D (CYPD) (141). This dysregulation results in impaired mitochondrial Ca²^+^ uptake and destabilization of membrane potential. Depletion of mtDNA or knockdown of SPG7/AFG3L2 suppresses IL-1β maturation, confirming that PHB1 deficiency drives inflammation through a mitochondrial stress–dependent, NLRP3-inflammasome pathway. Elevated ROS in PHB1-deficient cells further contributes to inflammasome activation, though mtDNA appears to be the main trigger. Collectively, these findings reveal that PHB1 maintains mitochondrial integrity via the m-AAA protease to restrain mtDNA-dependent inflammasome activation under stress conditions (141).

AFG3L2 can also modulate intestinal inflammation, particularly in inflammatory diseases such as Crohn’s disease. In the immune cell deconvolution analysis, Crohn’s disease tissues demonstrate an increase in infiltration of plasma cells and neutrophils but reduced levels of follicular helper T cells and eosinophils. AFG3L2 expression was negatively correlated with neutrophil infiltration and positively correlated with eosinophil infiltration, indicating that reduced AFG3L2 levels may promote neutrophil-driven inflammation while limiting eosinophil-associated immune regulation (147). Functional assays confirmed that AFG3L2 overexpression in intestinal epithelial cells mitigated LPS-induced inflammatory damage by enhancing cell viability, reducing LDH leakage, restoring tight junction proteins (ZO-1 and occludin), and upregulating antioxidant defenses (NRF2 and HO-1) in NCM460 cells (147). Ferroptosis is an iron-dependent form of regulated cell death driven by toxic lipid peroxidation (148), and its suppression may ameliorate ulcerative colitis (149). AFG3L2 can inhibit ferroptosis by restoring GPX4 and ferritin expression and suppressing ACSL4. This protection was mediated through activation of PPARα, as AFG3L2 promoted PPARα expression and nuclear translocation, leading to transcriptional upregulation of GPX4. Overexpression of *PPARA* mimicked AFG3L2’s anti-inflammatory and antioxidant effects, whereas *PPARA* silencing abolished them, confirming that the AFG3L2–PPARα–GPX4 axis is essential for epithelial protection. *In vivo*, overexpression of AFG3L2 ameliorated disease severity in a Crohn’s disease mouse model, reducing IL-1β and TNF-α levels, improving antioxidant balance, preserving mitochondrial ultrastructure, and decreasing immune cell infiltration in intestinal tissue. These results demonstrate that AFG3L2 preserves mitochondrial homeostasis and intestinal immune equilibrium by activating PPARα-dependent transcriptional responses that enhance GPX4 expression, suppress ferroptosis, and modulate immune infiltration patterns associated with intestinal inflammation (147).

Overall, m-AAA protease integrates multiple layers of mitochondrial quality control, OPA1 processing, preventing the accumulation of misfolded proteins, and mPTP regulation, to prevent the release of pro-inflammatory mitochondrial signals.

## 5 i-AAA protease, YME1L

YME1L is a mitochondrial inner-membrane AAA+ protease that plays a pivotal role in maintaining mitochondrial protein import, mitochondrial morphology, and cellular metabolism (150, 151). Functionally, YME1L regulates mitochondrial morphology through the selective degradation of GTPases, such as OPA1, and inner membrane proteins, which can trigger mitochondrial dynamics and stress responses (73, 152). It also modulates nucleotide homeostasis and pyrimidine pools, linking mitochondrial proteolysis to metabolic signaling and innate immune activation (153, 154). Like other AAA+ proteases, YME1L requires an accessible, unstructured segment to initiate engagement. After the unfolded substrate is recognized, the ATPase domain facilitates the translocation through a hydrophobic central pore into the hydrophilic proteolytic chamber. Compared to m-AAA, YME1L shows clearer evidence of sequence-dependent recognition, including a phenylalanine-rich motif (155). Key substrates of YME1L include *COX2* (156), *TIM10* (157), TIMM17A (151), TIMM23 (151), and UPS1 (158).

Activation of YME1L can be triggered by various insults; hypoxia and lipid signaling have been shown to drive its proteolytic activity (152, 159), while oxidative stress and nutrient imbalances also influence YME1L levels and activity (160). Oxidative stressors such as hydrogen peroxide (H_2_O_2_), paraquat, and arsenic lead to the degradation of YME1L in various cell types (SHSY5Y, HEK293T, N2a, HeLa). This degradation occurs independently of mitophagy, suggesting that the loss of YME1L is associated with stress-induced factors. The stability of YME1L is compromised due to ATP depletion following oxidative stress, which causes a loss of nucleotide binding to YME1L, making it more susceptible to degradation (160). This leads to the activation of the OMA1 protease, which plays a key role in degrading YME1L during oxidative damage. ATP supplementation partially rescues the degradation of YME1L, indicating that ATP levels are critical for maintaining YME1L’s stability. The degradation of YME1L under oxidative stress impairs its ability to regulate mitochondrial protein quality control, particularly the stability of proteins involved in mitochondrial function, like the TIM23 complex (160).

YME1L has been shown to play a role in the regulation of inflammation in renal tubular epithelial cells (RTECs) during diabetic kidney disease (DKD). Overexpression of YME1L in renal tubules of diabetic mice was shown to reduce the expression of inflammatory cytokines, including TGF-β, IL-6, IL-1α, and TNF-α (161). *In vitro*, this was associated with reductions in ROS production, increased ATP production, and enhanced mitophagy. Interestingly, Co-IP experiments demonstrate a physical interaction between YME1L and the mitophagy receptor BCL2L13, and siRNA knockdown of BCL2L13 prevented the increases in mitophagy seen with YME1L overexpression, suggesting this interaction functionally increases mitophagy. These results indicate YME1L as a regulator of mitochondrial homeostasis and inflammatory control in RTECs, with YME1L deficiency driving mitochondrial dysfunction, senescence, and SASP-mediated inflammation in DKD (161).

Similar to the other proteases discussed, in YME1L-deficient MEFs, cytosolic mtDNA accumulates and drives the activation of IFN response, marked by the upregulation of ISGs such as STAT1, cGAS, STING, and RIG-I. The depletion of mtDNA using 2′, 3′-dideoxycytidine (ddC) or ethidium bromide strongly suppressed ISG expression, demonstrating that the immune activation is likely dependent on mtDNA release rather than a direct effect of YME1L on the signaling machinery. mtDNA accumulates in the cytosol of YME1L-deficient cells, suggesting that YME1L contributes to mitochondrial homeostasis and indirectly prevents mtDNA release, thereby promoting an anti-inflammatory effect. Furthermore, SLC25A33, a mitochondrial pyrimidine carrier, accumulates in YME1L-deficient cells and is essential for mtDNA release and the activation of the cGAS–STING pathway, which was a secondary outcome of the dysfunction of pyrimidine metabolism and glutaminolysis, disrupting the mitochondrial homeostasis (153). Genetic or pharmacological restoration of pyrimidine nucleotide pools suppressed ISG expression, further supporting the notion that mtDNA release is an indirect outcome of mitochondrial stress caused by YME1L loss (153). In another study with YME1L-deficient cells, they also demonstrate impaired mtDNA replication, characterized by decreased synthesis and increased incorporation of ribonucleotides (rNMPs) into mtDNA, causing genomic instability. This instability, coupled with mtDNA damage, triggered the cGAS–STING–TBK1 immune pathway, leading to an inflammatory response marked by ISG expression. Additionally, the study shows that nucleotide imbalance, with an elevated Ribonucleoside triphosphate (rNTP) to deoxyribonucleoside triphosphate (dNTP) ratio, can further exacerbate mtDNA damage and immune activation. Importantly, restoring nucleotide balance in these cells suppressed the immune response, underscoring the potential role of nucleotide homeostasis in regulating immune activation related to mtDNA instability (154).

By maintaining protein quality control and nucleotide homeostasis, YME1L preserves mitochondrial function and prevents the release of mitochondrial DAMPs that trigger innate immune activation. Dysregulation or loss of YME1L, especially under oxidative or metabolic stress, leads to mitochondrial dysfunction, mtDNA instability, and activation of pro-inflammatory pathways including cGAS–STING. In addition, YME1L regulates OPA1, together with OMA1; therefore, this part of YME1L’s regulation of inflammation is discussed separately.

## 6 OMA1

OMA1 is a zinc metalloprotease located in the inner mitochondrial membrane that plays a key role in maintaining mitochondrial integrity under stress conditions. Unlike constitutively active mitochondrial proteases such as YME1L, OMA1 is dormant under basal conditions and becomes rapidly activated in response to mitochondrial dysfunction, including loss of membrane potential, oxidative stress, or proteostatic imbalance (162–165). Upon activation, OMA1 cleaves the dynamin-like GTPase OPA1, converting it from its L-OPA1, fusion-competent form to S-OPA1, fusion-inactive isoforms, thereby promoting mitochondrial fragmentation (165). Given that mitochondrial dynamics and metabolism are central to immune cell activation, OMA1-mediated mitochondrial fragmentation may influence inflammatory responses by altering metabolic flux and stress signaling, though this remains to be fully elucidated. The mechanism that OMA1 recognizes and cleaves its substrates remains incompletely understood. While OMA1 is known to be activated by stress-induced changes in mitochondrial membrane potential, the role of the N-terminal sensor domain in sensing these changes and triggering substrate cleavage is not yet fully clarified. This domain, located on the matrix side of the inner membrane, is crucial for proteolytic activation (164), but how it detects membrane potential fluctuations and subsequently activates OMA1’s proteolytic activity on substrates remains unclear (166). The direct substrates of OMA1 include; DELE1, which activates the integrated stress response (167, 168), PINK1, which is involved in mitophagy (169, 170), PGAM5, a serine/threonine protein phosphatase that plays a role in the regulation of the mitochondrial fission protein DRP1 (171, 172), as well as autocleavage of OMA1 upon activation (162). Under basal conditions, OMA1 cleaves and destabilizes PINK1, thereby suppressing mitophagy initiation. Loss or inhibition of OMA1 stabilizes PINK1 on the outer mitochondrial membrane, promoting mitophagy activation (169, 170). These downstream effects highlight that the OMA1 activity influences mitochondrial quality control and cellular stress responses.

Under conditions of mitochondrial dysfunction, OMA1 cleaves the inner membrane protein DELE1, enabling its translocation to the cytosol where it activates the eIF2α kinase HRI. This leads to eIF2α phosphorylation and subsequent ATF4-dependent transcriptional reprogramming (173). Downstream, activation of this pathway promotes antioxidant defense, metabolic rewiring, and protection against ferroptotic cell death, as observed in cardiomyocytes. Loss of OMA1 disrupts this signaling axis, impairing ISR, increasing lipid peroxidation, and promoting ferroptotic cell loss (173). Beyond cardiomyocytes, ATF4 plays an important role in macrophages, T cells, B cells, NK cells and dendritic cells (174–176). However, whether ATF4 promotes or attenuates inflammatory cytokine production is context-dependent, with studies showing both pro- and anti-inflammatory effects of genetic manipulation of ATF4 (176–178). Additionally, ATF4 plays a critical role in regulating amino acid metabolism during T cell activation (179, 180). In preclinical studies, the OMA1 activator, BTM-3566, has been shown to activate the OMA1-DELE1-ATF4 stress pathway to drive apoptosis in tumor cells. Importantly, these effects are specific to OMA1, as CRISPR–Cas9 depletion of OMA1 eliminates BTM-3566’s apoptotic activity (47). BTM-3566 is currently under investigation in a phase 1 clinical trial for the treatment of B-cell lymphomas (48).

In addition to proteolytic degradation of target proteins, OMA1 has also been shown to influence cell signaling through binding (without degradation) to proteins, including inositol 1, 4, 5-trisphosphate receptor (IP3R) and heat shock protein 9 (HSPA9). This binding disrupts the formation of the IP3R/HSPA9/VDAC1 complex at mitochondria-associated membranes, a structure that normally maintains mitochondrial integrity and regulates mitochondrial–endoplasmic reticulum communication. By preventing assembly of this complex, OMA1 may contribute to facilitating mitochondrial outer membrane permeabilization and the subsequent release of mtDNA into the cytosol. The inhibition of mitophagy with Mdivi-1 reduced cytosolic mtDNA release and attenuated cGAS–STING activation, indicating that mtDNA translocation is mediated indirectly through OMA1-induced mitophagy which can trigger type I interferon production and downstream inflammatory signaling. Additional RNAseq studies have found that Oma1^-/-^ Parkin^-/-^ mice display an increase in immune-related genes through GSEA analysis, with 9 of the top 10 GSEA hits being related to immune processes. This was associated with increases in ISG expression and mtDNA-mediated activation of the cGAS-STING pathway (181).

Together, these studies identify OMA1 as a central mediator linking mitochondrial stress to cellular adaptation and immune signaling. Through both proteolytic and non-proteolytic mechanisms, OMA1 coordinates mitochondrial quality control, activates stress pathways such as DELE1–ATF4, and promotes innate immune signaling via cGAS–STING. Its emerging roles in immunity and disease highlight OMA1 as a potential therapeutic target for conditions driven by mitochondrial dysfunction and inflammation.

## 7 OPA1

OPA1 is located in the intermembrane space and is anchored to the inner mitochondrial membrane. It is the primary substrate of OMA1 and is also regulated by YME1L and the m-AAA protease; their functional interplay defines mitochondrial dynamics under stress. Cleavage of OPA1 not only determines mitochondrial morphology but also impacts respiratory efficiency, mtDNA release, and inflammatory signaling. OPA1 is critical in the regulation of mitochondrial fusion and fission. OPA1 is synthesized with an N-terminal mitochondrial targeting sequence and cleaved by the mitochondrial processing peptidase after the protein enters the mitochondrial matrix. After passing through the TIM complex, OPA1 adopts an L-OPA1 isoform that anchors in the inner mitochondrial membrane (182). A fraction of L-OPA1 undergoes proteolytic cleavage by the inner-membrane proteases OMA1 and YME1L at the S1 and S2 sites, respectively, producing S-OPA1 isoforms. The balance of L-OPA1 and S-OPA1 is crucial for mitochondrial fusion, with the S-OPA1 isoforms regulating fusion activity (183). Also, the L-OPA and S-OPA isoforms are involved in non-fusion roles, such as maintaining mitochondrial DNA and respiratory chain supercomplexes, demonstrating that the OPA1 cleavage plays a central role in mitochondrial dynamics (184). Given that immune cell activation and function are tightly linked to mitochondrial morphology and metabolism (185), OPA1-dependent control of mitochondrial dynamics is a key regulator of inflammatory and immune responses, as discussed below.

OMA1-dependent cleavage of OPA1 has recently been highlighted as important in T-cell acute lymphoblastic leukemia (T-ALL). Induction of mtROS with inhibition of the pentose phosphate pathway selectively killed glucocorticoid-resistant T-ALL cells by triggering OMA1-dependent cleavage of OPA1. siRNA knockdown of OMA1 confirmed the importance of this pathway in the mitochondrial fission and sensitization to apoptosis (186). In contrast to OMA1-driven OPA1 cleavage promoting apoptosis sensitivity in T-ALL, OPA1 upregulation in acute myeloid leukemia (AML) cells promotes mitochondrial cristae tightening, thereby restricting *cytochrome c* release and conferring resistance to apoptosis induced by BCL-2 inhibition. Pharmacological inhibition of OPA1 reversed this phenotype, restoring apoptotic sensitivity and revealing a metabolic dependency on glutamine oxidation that rendered cells susceptible to ferroptosis (187). While these studies were conducted in malignant cells, the results demonstrate that the OMA1/OPA1 pathway can function as a redox-sensitive effector coupling mitochondrial stress to metabolic and apoptotic remodeling. Additionally, glutamine dependency is also a trait of immune cells; for example, macrophages exhibit context-dependent glutamine dependency during inflammation, as glutamine metabolism supports both pro-inflammatory signaling through succinate accumulation and anti-inflammatory polarization via α-ketoglutarate-mediated epigenetic regulation (188).

In non-malignant cells, OPA1 cleavage is increased in response to LPS in alveolar macrophages, suggesting increased OMA1 activity during inflammation (189). Macrophages lacking OPA1 exhibited impaired TCA cycle function, characterized by the accumulation of intermediates such as succinate and α-ketoglutarate, leading to a disrupted balance between M1 and M2 macrophage polarization (190). In the myocardium, TLR4 activation is associated with reductions in OPA1. Interestingly, in this tissue, OMA1 levels were increased with TLR4 activation, while YME1L levels were decreased. These effects of LPS on OPA1/OMA1 and YME1L appear to be mediated by the downstream inflammatory signals, including TNFα and ROS production, when tested *in vitro (*191). Interestingly, treatment with TNFα and H_2_O_2_ resulted in mitochondrial damage, whereas IL1β and IL6 had no effect on mitochondrial structure or fragmentation (191), consistent with what others have seen in adipocytes (192). Using both OMA1 knockdown and YME1L overexpression, both proteases were found to be involved in OPA1 processing in response to TNFα and H_2_O_2_, and mitochondrial ultrastructure changes, which were associated with fragmented mitochondria, as expected.

The Jak2/STAT3 cascade in macrophages has also been shown to suppress OPA1 protein expression, leading to increased mitochondrial fragmentation, mtDNA release in the cytosol and activation of the cGAS/STING pathway (193). Whether this reduction in OPA1 expression involves increased activity of OMA1, YME1L, or the m-AAA protease, which regulates OPA1, has not been investigated. In HUVECs lacking OPA1, NFκB activity was increased and remained elevated despite VEGF treatment. This was associated with increased IκBα degradation in *Opa1-silenced* HUVECs (69). Mechanistically, this was associated with changes to Ca^2+^ signaling, as OPA1 interacts with the mitochondrial calcium uniporter. Indeed, OPA1 deletion led to an increase in cytosolic Ca^2+^ levels, which was associated with the increased NFκB activation, as these effects were corrected with a calcium chelator in the *Opa1*-silenced cells (69).

In other models of inflammation, such as Hypoxic-ischemic brain injury (HIBD), OPA1 cleavage has also been shown to be increased. Interestingly, the suppression of OPA1 expression with small molecule MYLS22 exacerbates inflammation in HIBD, resulting in increased expression of the NLRP3 inflammasome components, including NLRP3, ASC, GSDMD, Caspase-1, IL-18 and IL-1β. Conversely, overexpression of L-OPA1 attenuates the expression of inflammasome components and inflammasome activation (49). Interestingly, increased OPA1 expression is also associated with macrophage infiltration in breast cancer tumors (194). In contrast to the inhibitory effects of MYLS22 on OPA1, the type II diabetes medication, Empagliflozin, which is an SGLT2 inhibitor that decreases glucose levels by inhibiting glucose reabsorption in the renal tubule, has been shown to reduce OPA1 cleavage, enhancing mitochondrial fusion and neutralizing mtROS production in the BV2 microglial cell line. Notably, genetic ablation of OPA1 abolished the anti-inflammatory effects of empagliflozin in this model, highlighting the essential role of OPA1 in mediating therapeutic effects (51). While the effects of empagliflozin on OPA1 are indirect, this highlights mitochondrial dynamics as a key downstream effector of its anti-inflammatory mechanism.

Collectively, these findings highlight OPA1 as a critical regulator of immune cell metabolism and inflammatory signaling. By integrating cues from OMA1, YME1L and m-AAA protease, OPA1 controls mitochondrial fusion, respiratory efficiency, and mtDNA release, processes that directly influence cytokine production, inflammasome activation, and macrophage polarization. Dysregulation of OPA1 disrupts metabolic balance and promotes aberrant activation of pathways such as NFκB and cGAS–STING, linking mitochondrial stress to innate immune activation. Thus, OPA1 serves as a key mitochondrial effector coupling bioenergetic state to immune cell function and inflammatory outcomes.

## 8 Discussion

Mitochondrial proteases have emerged as central regulators of innate immunity, integrating mitochondrial stress, protein quality control, and metabolic adaptation into coordinated inflammatory responses. Across diverse immune and non-immune cell types, mitochondrial proteases such as LONP1, CLPXP, AFG3L2/SPG7, YME1L, and OMA1 can shape the balance between mitochondrial homeostasis and the activation of stress pathways that drive cytokine production, type I interferon signaling, and inflammatory cell fate regulation.

A recurring theme across studies is that mitochondrial proteases couple mitochondrial dysfunction to inflammatory signaling through three major axes*: the integrated stress response (ISR), mtDNA-driven innate immune pathways, and oxidative stress.* Under normal conditions, LONP1 degrades newly synthesized DELE1 following mitochondrial import (108), thereby restraining DELE1-HRI-ISR activation. In contrast, mitochondrial stress activates OMA1, which directly cleaves DELE1 to generate cytosolic fragments and activates HRI. This leads to eIF2α phosphorylation and subsequent ISR activation linking mitochondrial protein stress to transcriptional reprogramming in macrophages, T cells, and stromal cells. Additionally, ATF4, activated through these pathways, regulates amino acid metabolism during T cell activation, further linking mitochondrial stress to immune cell function and inflammation (179, 180). Indeed, the role of ATF4 in immunity and inflammation has been extensively studied, and demonstrates that these proteases can link mitochondrial stress to ATF4 activation, in the absence of typical ER stress pathways, which is an important mechanistic link between mitochondria, ATF4 and immune cell function.

Parallel to this, proteases, including LONP1, OMA1, YME1L, and CLPXP, govern mtDNA stability, nucleoid maintenance, and mitochondrial membrane integrity. Their dysfunction may promote cytosolic mtDNA accumulation, thereby activating cGAS–STING, RIG-I/MAVS, and downstream interferon pathways that contribute to autoimmunity, antiviral immunity, and tumor immunogenicity. LONP1 plays a role in mtDNA maintenance by binding the DNA and interacting with other protein nucleoid components implicated in mitochondrial transcription (195), such as TFAM. How LONP1 regulates mtDNA release, whether it is associated with TFAM degradation, and therefore disrupted nucleoid organization, and mtDNA packaging, or as a secondary consequence, mitochondrial disruption is still unclear. Similarly, ClpP KO cells exhibit enlarged and aggregated mtDNA nucleoids, suggesting a role for ClpP in mtDNA integrity (86); however, it’s still unclear whether ClpP directly regulates mtDNA containment or if the observed mtDNA release is an indirect consequence of nucleotide imbalance. Other proteases can also influence mtDNA leakage; however, how these mechanisms are regulated, such as through the mPTP, VDAC, and BAK-Bax pores, warrants further investigation. AFG3L2 interaction with SPG7, leads to the pathological opening of the mPTP. OMA1, similarly, facilitates mtDNA release by destabilizing the mitochondrial outer membrane. Defining how mitochondrial proteases intersect with these membrane-permeabilizing pathways will be essential for understanding the full spectrum of mtDNA-driven inflammatory signaling.

Finally, proteases such as OMA1, LONP1, AFG3L2, and YME1L respond to changes in mitochondrial redox state, tuning ROS production, cristae architecture, and susceptibility to cell death pathways, including apoptosis and ferroptosis. Oxidative stressors like hydrogen peroxide (H_2_O_2_) and paraquat induce the degradation of YME1L, impairing its role in mitochondrial quality control. This degradation occurs independently of mitophagy, suggesting that oxidative stress directly destabilizes YME1L, compromising its ability to maintain mitochondrial function (160). AFG3L2 overexpression reduced oxidative stress, preserved mitochondrial structure, and alleviated inflammation in murine models of Crohn’s disease, highlighting its role in protecting against ROS-induced damage (166).

A central and unresolved challenge in this field is explaining why the same mitochondrial protease can be pro-inflammatory in one context and anti-inflammatory in another. The studies reviewed here suggest that this apparent contradiction reflects the integration of several intersecting variables. First, cell lineage and proliferative state are critical determinants: in rapidly dividing cancer cells with high proteostatic demand, protease activation or loss tends to overwhelm mitochondrial homeostasis, triggering cytotoxic stress and immune activation, whereas in postmitotic cells such as neurons or quiescent immune cells, the same perturbation unfolds more chronically and through distinct stress pathways. Second, the degree of pre-existing mitochondrial dysfunction shapes the response; in cells with already-compromised mitochondria, such as in mitochondrial encephalopathies or NASH, loss of a protease like ClpP or restoration of LONP1 can paradoxically be protective by reducing the burden on a dysfunctional system. Third, mitophagy competence and OxPhos dependence set the threshold at which mitochondrial dysfunction is tolerated before danger signals are released. Finally, it matters whether the dominant phenotype arises from loss of housekeeping function, which leads to gradual mtDNA instability and sterile inflammation, versus acute stress-pathway engagement, which can rapidly activate or suppress immune outputs. Overall, these determinants suggest that the inflammatory output of mitochondrial protease perturbation is best understood not as a fixed property of the protease but as a property of the cell’s metabolic state and capacity for mitochondrial quality control.

Importantly, several small-molecule inhibitors and activators of these mitochondrial proteases have been tested for anti-cancer effects. It is notable that many also have effects on inflammation and immunity (**Table 1**), which could provide potential repurposing of failed chemotherapeutics for the treatment of infections and inflammatory diseases. However, the translational potential of these agents must be interpreted cautiously. Most compounds discussed here are mixed-action molecules with limited disease-selective validation, and few have been developed or optimized specifically for inflammatory indications. Indeed, CDDO-Me may be the most advanced example of this, developed for the treatment of blood cancers, tested for metabolic diseases where it had potent anti-inflammatory effects, while having some safety issues (long-term increase in heart failure), these agents may be effective as acute treatments for inflammatory conditions or to manage flare-ups of autoimmune diseases. Conversely, A54556A restores mitochondrial function in NASH, lowering ROS, suppressing inflammation, and improving liver pathology (57) but remains an early-stage probe without established selectivity profiling. Together, these compounds are best regarded as chemical tools that provide proof-of-concept for the broader hypothesis that mitochondrial proteostasis shapes inflammatory outcomes, rather than as validated therapeutic leads. Development of selective, protease-specific modulators with well-defined mechanisms will be essential before the therapeutic potential of this target class can be meaningfully evaluated in inflammatory disease.

Overall, mitochondrial proteases sit at a critical intersection between metabolism, stress adaptation, and immunity. Continued investigation into their substrates, regulatory networks, and context-dependent functions will not only deepen our understanding of immune–metabolic crosstalk but also guide the development of next-generation therapies aimed at restoring immune homeostasis in chronic inflammatory disease, cancer, and metabolic disorders.
